# Error-prone DnaE2 Balances the Genome Mutation Rates in *Myxococcus xanthus* DK1622

**DOI:** 10.3389/fmicb.2017.00122

**Published:** 2017-02-01

**Authors:** Ran Peng, Jiang-he Chen, Wan-wan Feng, Zheng Zhang, Jun Yin, Ze-shuo Li, Yue-zhong Li

**Affiliations:** State Key Laboratory of Microbial Technology, School of Life Science, Shandong UniversityJinan, China

**Keywords:** *dnaE2* gene, dispensable, error-prone, chromosome replication, growth, development and sporulation, *Myxococcus xanthus*

## Abstract

*dnaE* is an alpha subunit of the tripartite protein complex of DNA polymerase III that is responsible for the replication of bacterial genome. The *dnaE* gene is often duplicated in many bacteria, and the duplicated *dnaE* gene was reported dispensable for cell survivals and error-prone in DNA replication in a mystery. In this study, we found that all sequenced myxobacterial genomes possessed two *dnaE* genes. The duplicate *dnaE* genes were both highly conserved but evolved divergently, suggesting their importance in myxobacteria. Using *Myxococcus xanthus* DK1622 as a model, we confirmed that *dnaE*1 (*MXAN_5844*) was essential for cell survival, while *dnaE*2 (*MXAN_3982*) was dispensable and encoded an error-prone enzyme for replication. The deletion of *dnaE*2 had small effects on cellular growth and social motility, but significantly decreased the development and sporulation abilities, which could be recovered by the complementation of *dnaE*2. The expression of *dnaE*1 was always greatly higher than that of *dnaE*2 in either the growth or developmental stage. However, overexpression of *dnaE*2 could not make *dnaE*1 deletable, probably due to their protein structural and functional divergences. The *dnaE*2 overexpression not only improved the growth, development and sporulation abilities, but also raised the genome mutation rate of *M. xanthus*. We argued that the low-expressed error-prone *DnaE*2 played as a balancer for the genome mutation rates, ensuring low mutation rates for cell adaptation in new environments but avoiding damages from high mutation rates to cells.

## Introduction

DNA polymerase III is an enzyme complex responsible for prokaryotic genome replication (Kelman and O'donnell, 1995). The holoenzyme consists of a DNA polymerase (polymerase III α-subunit, PolIIIα), a processivity factor β-clamp and a clamp loader protein (McHenry, 2011a,b; Robinson et al., 2012). There are two types of PolIIIα: *dnaE* and PolC, both of which belong to the C-family of DNA polymerase (Ito and Braithwaite, 1991). While PolC exists in low-GC Gram-positive bacteria such as *Bacillus subtilis, dnaE* is universally distributed in different kinds of bacterial cells (Evans et al., 2008). The *dnaE* gene is essential in bacteria, and its functions have been well elucidated in some bacteria, such as *Escherichia coli* (Lamers et al., 2006). In addition to the essential *dnaE* gene, many bacterial species have evolved a second *dnaE* copy, usually named as *dnaE2*. For example, duplicated *dnaE* genes are often existed in those bacteria possessing large-sized genomes with high G+C contents (Zhao et al., 2007). *dnaE2* normally exists in a three-gene operon in Proteobacteria, accompanying with *imuA* and *imuB*, and the operon is regulated by LexA2, a possible transcriptional regulator associated with DNA damage response (Abella et al., 2004). Previous genetic studies indicated that *dnaE2* was non-essential for the chromosomal DNA replication in bacterial cells (Boshoff et al., 2003; Abella et al., 2004; Warner et al., 2010; Tsai et al., 2012). In *Streptomyces*, DnaE2 was reported to be an error-prone enzyme, associating with the DNA damage-inducible translesion DNA synthesis (Tsai et al., 2012). In *Mycobacterium tuberculosis*, the error-prone DnaE2 was proved *in vitro* to act as a primary mediator for cell survival through inducing mutagenesis and thus contributing directly to the emergence of drug resistance (Boshoff et al., 2003). However, this function is not in prevalence. For example, researches in *Pseudomonas* and *Streptomyces* indicated that *dnaE2* was not required for replication, end patching, or ultraviolet resistance and mutagenesis (Koorits et al., 2007; Tsai et al., 2012). The role of the replicated DnaE2 remains mostly unclear yet.

Myxobacteria are phylogenetically located in the delta division of the Proteobacteria (Shimkets et al., 2006). The bacteria are widely distributed in various environmental conditions, playing as micropredators by feeding on other microbial cells or macromolecules (Reichenbach, 1999; Jiang et al., 2010; Brinkhoff et al., 2012; Li et al., 2012; Zhou et al., 2014). Myxobacteria are characterized among the Prokaryotes by their complex multicellular social behaviors: cells locomote on solid surfaces in swarms to collaboratively prey on other microbial cells and, when food is scarce, aggregate to develop multicellular fruiting bodies, inside which differentiate metabolically quiescent myxospores (Shimkets, 1990; Kaiser and Losick, 1993; Dworkin, 1996; Shimkets et al., 2006). In this study, we bioinformatically analyzed those sequenced myxobacteria, and found that each of the myxobacterial genomes possessed two *dnaE* genes. The duplicate myxobacterial *dnaE* genes were both highly conserved but evolved divergently. Using *Myxococcus xanthus* DK1622 as a model, we confirmed that one *dnaE* gene was essential for cell survival, while the other was dispensable and error-prone. We evaluated functions of the *dnaE* genes and assayed their expressions in vegetative growth stage and developmental stage. It is known that there are two processes involving the genome replication in *M. xanthus*, one in the growth stage and the other in the early development stage (Tzeng and Singer, 2005). We found that the non-essential *dnaE* gene played functions in both of the two replication processes in *M. xanthus* DK1622. We suggested that the error-prone DnaE2 played as a balancer for the genome mutation rates via expression regulation, thus ensuring low mutation rates for the adaptation in new environments and avoiding high mutation rates to damage cells.

## Results

### *dnaE1* is essential while *dnaE2* is dispensable and encodes an error-prone DNA polymerase in *M. xanthus*

Myxobacterial genomes are normally larger than nine Mbp in size, but with some exceptions of approximate five-Mbp-genomes in *Anaeromyxobacter* strains. The G+C contents of the sequenced myxobacterial genomes ranged from 67.4% in *Enhygromyxa salina* DSM 15201 to 74.9% in *Anaeromyxobacter dehalogenans* 2CP-C. All the sequenced myxobacterial genomes, no matter in large size or small size, contained two *dnaE* genes (Table S1). Phylogenetic analysis indicated that the two protein sequences encoded by the duplicate myxobacterial *dnaE* genes were clustered separately with determined DnaE1 and DnaE2 of other bacteria, forming the DnaE1 and DnaE2 groups (Figure S1). The single DnaE gene of *E. coli* was in the DnaE1 group, clearly distant from the DnaE2 group; and the two DnaE-group trees had highly similar topologies. Consistent with the previous results (Timinskas et al., 2014; Wu et al., 2014), the emergence of myxobacterial *dnaE2* was derived from an early duplication event of the primordial *dnaE*. High conservation but great divergence suggested that the duplicate DnaE polymerases were both important for myxobacterial cells, but functioned divergently.

*M. xanthus* DK1622 is the model strain of myxobacteria. In the genome of DK1622, two genes, i.e., *MXAN_3982* and *MXAN_5844*, were predicted to be *dnaE*, encoding for the alpha subunit of DNA polymerase III (Goldman et al., 2006). The proteins encoded by the two *dnaE* genes contain 1185 and 1013 amino acids (MXAN_5844 and MXAN_3982), respectively. These two proteins were phylogenetically distant, having 48% similarity and 30% identity of their amino acid sequences. According to their phylogenetic locations (Figure S1), the *MXAN_3982* gene was designated as *dnaE2*, and the *MXAN_5844* gene was *dnaE1*.

**Figure 1:**
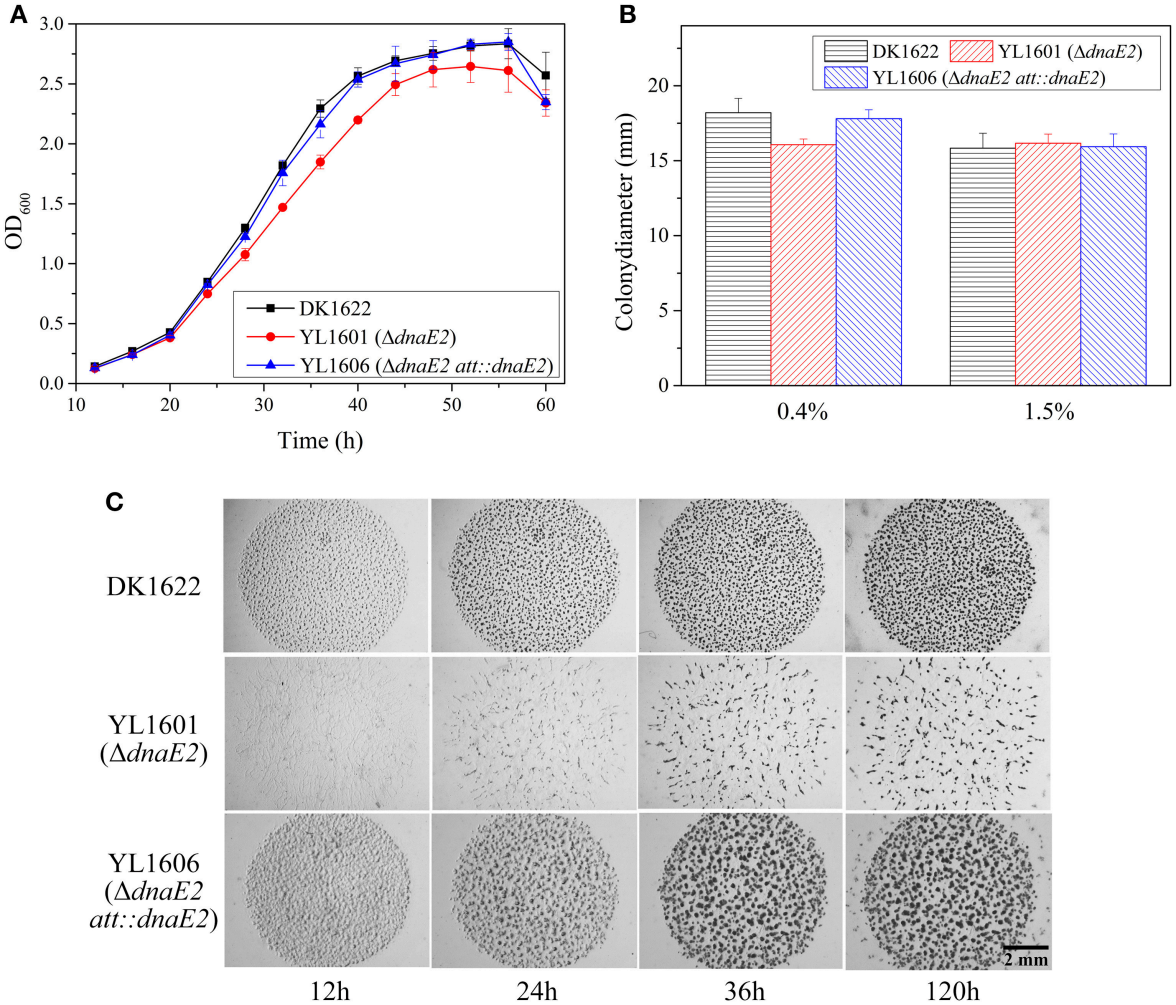
**Comparison of phenotypic characteristics of the *dnaE2* mutant (YL1601), the *dnaE2*-complementary mutant (YL1606) and the wild type strain DK1622. (A)** Growth curves of YL1601, YL1606, and DK1622 in CTT liquid medium. OD600 values were measured every 4 h until 60 h of incubation. The data were analyzed using two-way ANOVA. **(B)** Cellular swarming abilities on 0.4 and 1.5% agar CTT plate. **(C)** Aggregation and formation abilities of fruiting bodies on TPM development plate. The error bars represent the standard deviation of three independent experiments.

To evaluate their essentiality, we made deletion mutations of the *dnaE1* and *dnaE2* gene in *M. xanthus* DK1622, respectively. While *dnaE2* was deletable, producing the mutant of YL1601, the *dnaE1* gene could not be deleted, which was further confirmed by failed attempts to delete the *dnaE1* gene in a *dnaE2*-overexpressing mutant (see below). Compared with that of the wild-type strain DK1622, the OD values of the *dnaE2* deletion mutant YL1601 were smaller than the wild type at different time points during the exponential growth stage in CTT growth medium (two-way ANOVA, *P* < 0.01; Figure 1A). To confirm the function of *dnaE2* in growth, we further constructed a mutant by inserting the *dnaE2* gene following its own promoter at the *attB* site in the YL1601 genome, forming the YL1606 mutant. As expected, the complementary strain recovered the delayed growth in YL1601, showing almost the same growth curve as the wild type strain DK1622 (Figure 1A).

To confirm whether the DnaE2 protein was an error-prone enzyme, we constructed two more mutants by deleting two DNA-repair genes of *mutL* (*MXAN_4026*) and *mutS* (*MXAN_3897*) from DK1622, respectively, producing the mutants of YL1602 and YL1603. The wild type strain DK1622 and the three mutants of YL1601, YL1602 and YL1603 were cultivated on CTT growth medium supplemented with nalidixic acid (40 μg/ml) to evaluate their production abilities of resistant mutations. It is known that the inhibition of nalidixic acid on DNA repair can be reversed by the MutL and MutS enzymes of the DNA repairing system in *E. coli* cells, thus the deletion mutant of *mutL* or *mutS* will produce more genome mutations than the wild type strain during the chromosome replication process (Sniegowski et al., 1997). In *M. xanthus*, the mutation rate of DK1622 was approximately 3.69 × 10^−8^ per nucleotide in the presence of nalidixic acid; whereas the YL1602 and YL1603 mutants produced mutations of 5.41 × 10^−7^ and 1.77 × 10^−6^, respectively (Table 1). The mutation rates of the *mutL* and *mutS* mutants were both significantly higher than that of DK1622 (*t*-test, *P* < 0.01). This result was consistent with that reported in *E. coli* (Sniegowski et al., 1997), suggesting the method was also reliable in *M. xanthus* for the evaluation of mutation rates. As expected, the deletion of *dnaE2* made the mutant, possessing only *dnaE1*, to have nearly 10 times lower of mutation rate than that of the wild type strain DK1622, which harbors both *dnaE1* and *dnaE2* (Table 1). This result confirmed the error-prone characteristics of the DnaE2 protein in *M. xanthus* cells.

**Table 1:** **Mutation rates of *M. xanthus* DK1622 and mutants grown on CTT plates supplemented with nalidixic acid**.

**Strains**	**Cell num**.	**Average count**	**Mutation rate**	**St.dev**
DK1622	5.0 × 10^9^	185	3.69 × 10^−8^	2.05 × 10^−9^
YL1602 (Δ*mutS*)	5.0 × 10^9^	2707	5.41 × 10^−7^	5.05 × 10^−8^
YL1603 (Δ*mutL*)	5.0 × 10^9^	8842	1.77 × 10^−6^	2.24 × 10^−7^
YL1601 (Δ*dnaE2*)	5.0 × 10^9^	24	4.87 × 10^−9^	5.03 × 10^−10^
YL1604 (DK1622 *att::Kan*)	5.0 × 10^9^	214	4.28 × 10^−8^	2.05 × 10^−9^
YL1605 (DK1622 *att::dnaE2*)	5.0 × 10^9^	682	1.36 × 10^−7^	1.86 × 10^−8^

### Effects of the deletion of *dnaE2* on *M. xanthus* social behaviors

The above results indicated that *dnaE1* was essential for the survival of *M. xanthus* cells, while the error-prone DnaE2 gene was dispensable for cell survival. *dnaE2* also involved in cellular growth, but in weak effects and low fidelity. We further assayed effects of the deletion of *dnaE2* in the swarming and developmental processes in *M. xanthus*. Similar to the growth change, the swarming ability of the YL1601 mutant was weakly decreased on 0.4% CTT plate, comparing with that of DK1622 (*t*-test, *P* = 0.051), while the motility on 1.5% CTT plate showed no difference between DK1622 and YL1601 (Figure 1B).

When cultivated on the TPM developmental medium, cells of the *dnaE2* deletion mutant aggregated irregularly (Figure 1C). From the phenotypes of the mutant on TPM, the *dnaE2* gene probably played functions in the early stage of the aggregation in the development process. The sporulation ability of the *dnaE2* mutant was decreased to approximately 70% of that of DK1622 after 5 days of incubation on TPM medium (1.49 × 10^6^ ± 9.61 × 10^4^ vs. 2.14 × 10^6^ ± 1.70 × 10^5^; Table 2). If the *dnaE2* gene was complemented into the *dnaE2* deletion mutant (YL1606), the irregular aggregation phenotype on TPM developmental medium was completely recovered (Figure 1C). This result suggested that the DnaE2 protein also played functions in the early development stage of *M. xanthus* DK1622 cells. Seemly, the function of the *dnaE2* gene was more obvious in the development stage than its function in the growth stage.

**Table 2:** **Sporulation abilities of *M. xanthus* DK1622 and YL1601 (Δ*dnaE2*) cells grown on TPM plates treated with nalidixic acid for1 h at different time points**.

**Time of addition**	**Spore production in DK1622**	**Percentage of control%**	**Spore production in YL1601**	**Percentage of control%**
Control	2.14 × 10^6^ ± 1.70 × 10^5^	100	1.49 × 10^6^ ± 9.61 × 10^4^	100
0 h	1.52 × 10^5^ ± 9.60 × 10^3^	7.13	5.53 × 10^4^ ± 6.51 × 10^3^	3.72
6 h	1.45 × 10^6^ ± 1.11 × 10^5^	68.02	3.50 × 10^5^ ± 3.61 × 10^4^	23.54
12 h	2.12 × 10^6^ ± 1.31 × 10^5^	99.22	7.67 × 10^6^ ± 4.51 × 10^4^	51.57
18 h	2.21 × 10^6^ ± 1.99 × 10^5^	103.59	1.08 × 10^6^ ± 2.10 × 10^5^	72.65
24 h	2.33 × 10^6^ ± 1.21 × 10^5^	108.89	1.54 × 10^6^ ± 1.12 × 10^5^	103.81

*The sporulation ability was assayed after 5 days of incubation on TPM*.

### Functions of *dnaE2* in the chromosome replication progress in development

Tzeng and Singer found that there is a DNA replication process during the development of fruiting bodies and sporulation in *M. xanthus* DK1622 (Errington and Wake, 1991; Tzeng and Singer, 2005). Accordingly, there are two processes involving the chromosome replication during the lifecycle of *M. xanthus* cells: one in the growth stage and the other in the development stage. To determine DnaE2 function in development, we inoculated concentrated cells of DK1622 and YL1601 on the TPM plates, and exposed culture plates with nalidixic acid solution at the final concentration of 20 μg/ml for 1 h at different cultivation time points. After 120 h of incubation, the sporulation rates of the cultures were calculated and compared with that of their respective controls without the exposure of nalidixic acid. Consistent with the previous report (Tzeng et al., 2006), the developmental progress of DK1622 was arrested by nalidixic acid in the early stage of development. After 12 h of incubation, the nalidixic acid exposure had nearly no effect on the sporulation of *M. xanthus* DK1622 cells (Table 2). In contrast, the sporulation ability of the *dnaE2* deletion mutant YL1601 was lower than that of DK1622 after the nalidixic acid exposure at each time point. The developmental arrest in YL1601 by the exposure of nalidixic acid was released after 24 h, which was nearly two-times long of that in DK1622. This result indicated that the deletion of *dnaE2* markedly prolonged the process of chromosome replication in development. Thus, together with the *dnaE1* gene, the *dnaE2* gene involved in the two processes of chromosome replication during the lifecycle of *M. xanthus* cells; its function in the development progress seemed to be more significant.

### *dnaE1* expression is greatly higher than that of *dnaE2*

**Figure 2:**
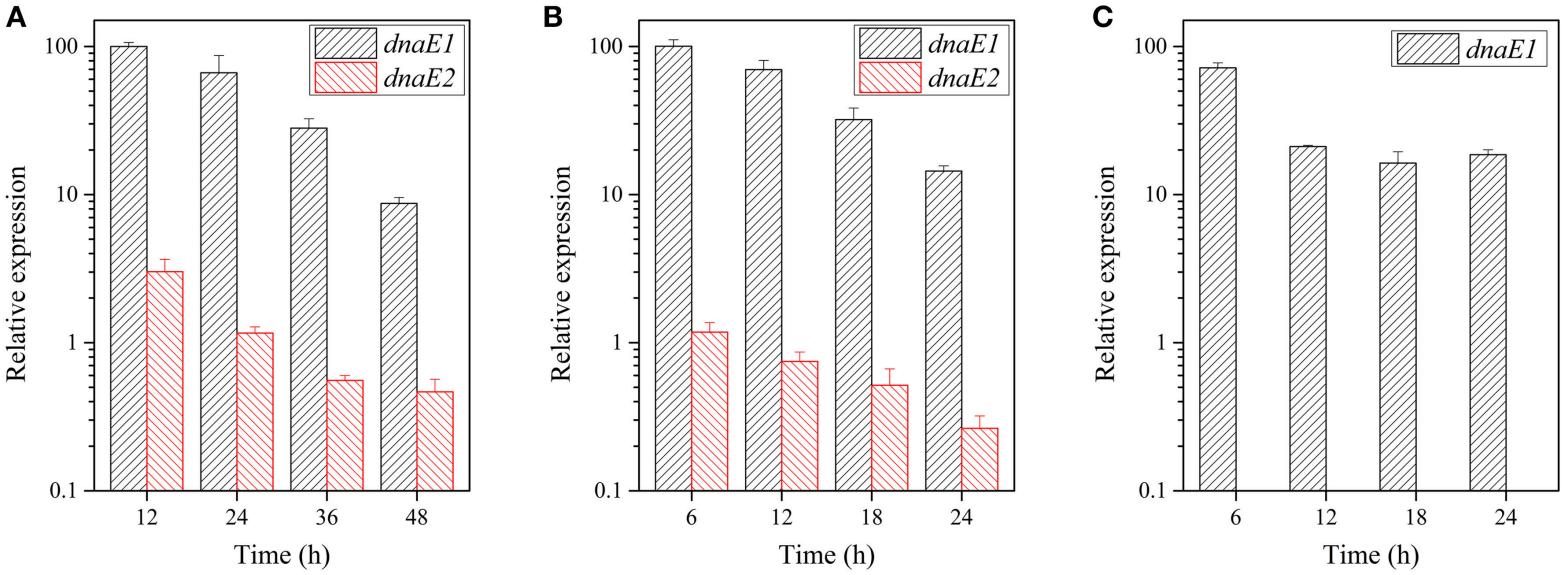
**Expression levels of the *dnaE1* and *dnaE2* genes at different time points under the nutritional and developmental conditions. (A)** *M. xanthus* DK1622 cells grown in the CTT growth medium. **(B)** The DK1622 strain grown on the TPM development plate. **(C)**. The YL1601 mutant cells grown on TPM plate. The expression of the *dnaE1* gene in CTT at 12 h of incubation was set as 100, and the others were the relative expressions. The error bars represent the standard deviation of three independent experiments.

There are two possible reasons for the differentiated functions of the *dnaE1* and *dnaE2* genes in the growth and developmental stages: structure differences and/or expression levels. We further assayed expression levels of the *dnaE1* and *dnaE2* genes under either the growth or the developmental conditions. Quantitative real-time PCR amplification showed that the expression levels of *dnaE1* were greatly higher than that of *dnaE2* at different time points under the CTT nutritional growth conditions (Figure 2A). For example, after 12 h of incubation in CTT liquid medium, the expression of *dnaE1* was more than 10 times of that of *dnaE2*. With the increase of incubation time, the expressed products of *dnaE1* were gradually decreased and reached to less than one-tenth (8.7%) of the 12-h-level at 48 h of incubation. Similarly, the expressions of *dnaE2* were also gradually decreased and were always significantly lower than that of *dnaE1* at each time point (*t*-test, *P* < 0.01).

The expressions of the two *dnaE*-genes were in similar patterns on the TPM development plate as they were in the growth conditions (Figure 2B). In contrast, the expressions of *dnaE2* in the developmental conditions were even lower than that in the growth conditions. After the deletion of *dnaE2*, no expression of *dnaE2* was detectable in the mutant, and the expression of the *dnaE1* gene was lower than that of the wild type strain at 6 h of incubation on the TPM plate (71.5%; Figure 2C). The results suggested that the deficiencies in the fruiting body formation and sporulation in the YL1601 mutant were probably resulted not only from the loss of *dnaE2*, but also from the decreased expression level of *dnaE1*.

### Overexpression of *dnaE2* improves growth, development, and mutation rate

**Figure 3:**
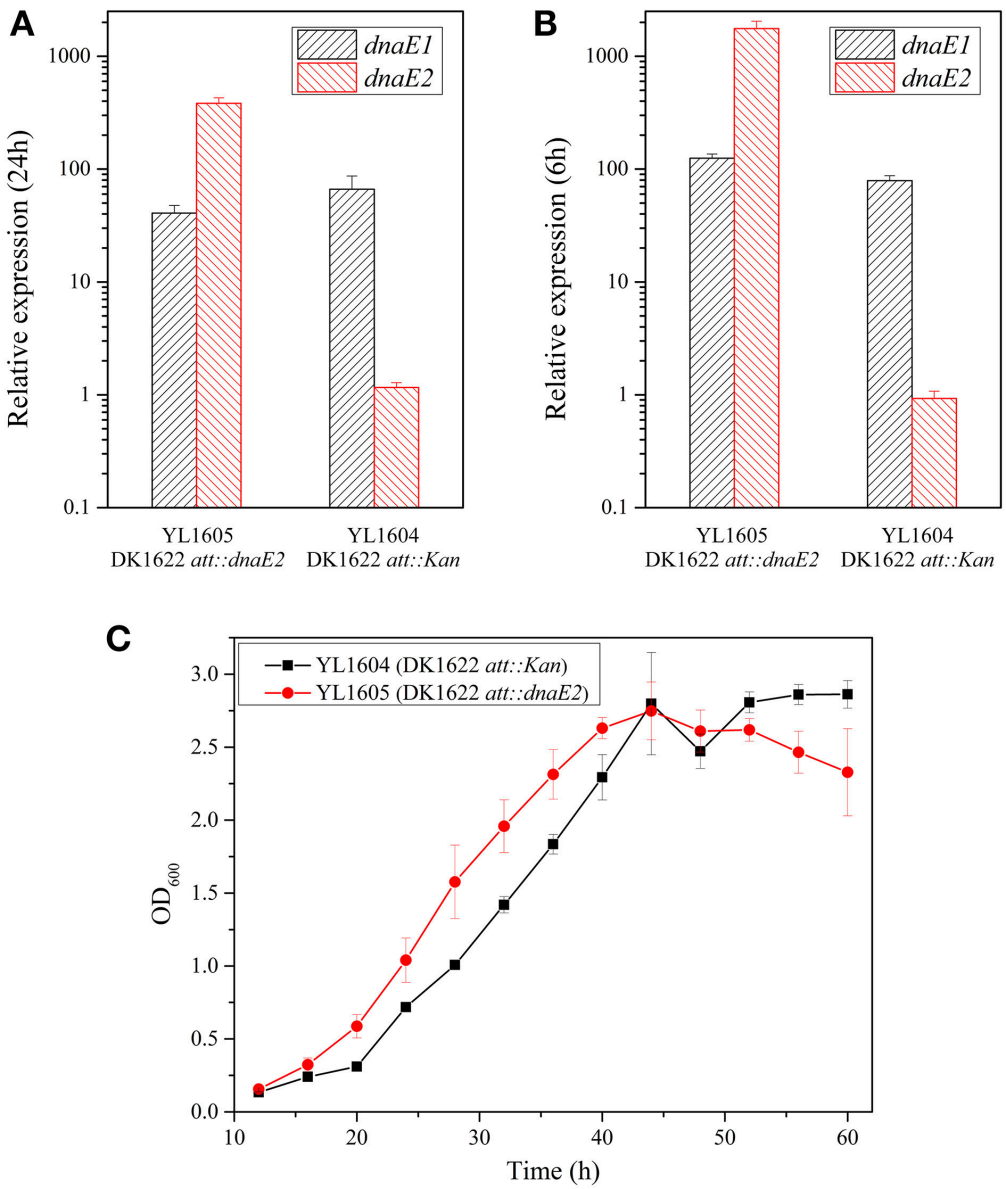
**Overexpression of the *dnaE2* gene in *M. xanthus* cells and cellular growth abilities. (A)** Expressions of *dnaE1* and *dnaE2* in YL1605 (DK1622 *att::dnaE2*) and YL1604 (DK1622 *att::Kan*) in CTT growth medium; **(B)** Expressions of *dnaE1* and *dnaE2* in the two mutants on TPM plate; **(C)** Growth curves of YL1605 and YL1604 in CTT liquid medium supplemented with kanamycin (40 μg/ml). OD600 values were measured every 4 h until 60 h of incubation. The data were analyzed using two-way ANOVA. The error bars represent the standard deviation of three independent experiments. The expressions of *dnaE1* and *dnaE2* in **(A)** and **(B)** of this Figure were the relative expressions, compared with the expression of the *dnaE1* gene in CTT at 12 h of nutritional condition of Figure 2A.

To further investigate effects of the *dnaE2* expression, we introduced a second copy of the gene into the *attB* site of DK1622 chromosome by the Mx8 integrase in pSWU19 (Wu and Kaiser, 1995), producing the YL1605 mutant (containing a local *dnaE2* and an introduced *dnaE2*). The introduced *dnaE2* gene was designed to follow the *pilA* promoter, a high efficient promoter in *M. xanthus* cells. Meanwhile, a mutant containing the empty pSWU19 plasmid was also constructed into the *attB* site of DK1622 chromosome as control (the mutant YL1604). The total expression levels of the two *dnaE2* genes were approximately 10 times higher than that of *dnaE1* in the YL1605 mutant under either the nutritional (Figure 3A; at 24 h of incubation in CTT; *t*-test, *P* < 0.01) or the developmental (Figure 3B; at 6 h of incubation on TPM; *t*-test, *P* < 0.01) conditions. In contrast, the expressions of *dnaE2* in YL1605 were approximately 500 times and 1500 times higher than that in YL1604 under the nutritional and the developmental conditions, respectively. These results suggested that the overexpression of *dnaE2* was mostly resulted from the introduced *dnaE2* gene. When cultivated in CTT liquid medium, the OD values of the *dnaE2* overexpression mutant YL1605 were higher than the control mutant YL1604 during the early exponential growth stage (Figure 3C). The growth differences were statistically significant (two-way ANOVA, *P* < 0.01). This result indicated that while the deletion of *dnaE2* led to weakly delayed growth, overexpression of the gene could weakly increase cellular growth.

Interestingly, the overexpression of *dnaE2* made the YL1605 mutant to have nearly three times higher of mutation rate than that of YL1604 cultivated on CTT plates supplemented with high concentrations of nalidixic acid (1.36 × 10^−7^ vs. 4.28 × 10^−8^, referred to Table 1). The difference was statistically significant (*t*-test, *P* < 0.01). In order to verify whether the overexpression changed the functions of *dnaE2* in the development progress, we exposed the TPM cultures of YL1605 and YL1604 mutants with nalidixic acid solution for 1 h at the same time points described above (Table 3). The results showed that the *dnaE2*-overexpressing mutant also had higher sporulation ability than the control when cells were treated with nalidixic acid in the early stage of development (before 12 h of incubation). Thus, overexpression of the *dnaE2* gene was not only able to increase the genome mutation rate, but also speed up the development progress. However, compared with effects of the *dnaE2* deletion, overexpression of *dnaE2* produced rather weak effects. The *dnaE1* gene still played major roles in the chromosome replication.

**Table 3:** **Sporulation abilities of *M. xanthus* YL1604 (DK1622 *att::Kan*) and YL1605 (DK1622 *att:: dnaE2*) cells grown on TPM plates treated with nalidixic acid for 1 h at different time points**.

**Time of addition**	**Spore production in YL1604**	**Percentage of control%**	**Spore production in YL1605**	**Percentage of control%**
Control	1.95 × 10^6^ ± 1.56 × 10^5^	100	2.10 × 10^6^ ± 1.81 × 10^5^	100
0 h	1.26 × 10^5^ ± 8.62 × 10^3^	6.43	1.93 × 10^5^ ± 8.74 × 10^3^	9.21
6 h	1.27 × 10^6^ ± 1.21 × 10^5^	65.02	1.83 × 10^6^ ± 1.56 × 10^5^	86.98
12 h	1.88 × 10^6^ ± 1.32 × 10^5^	96.25	2.09 × 10^6^ ± 1.85 × 10^5^	99.37
18 h	2.00 × 10^6^ ± 1.71 × 10^5^	102.56	2.13 × 10^6^ ± 1.50 × 10^5^	101.27
24 h	2.08 × 10^6^ ± 1.69 × 10^5^	108.89	2.21 × 10^6^± 2.01 × 10^5^	105.4

### Overexpression of *dnaE2* does not take place of *dnaE1*

**Figure 4:**
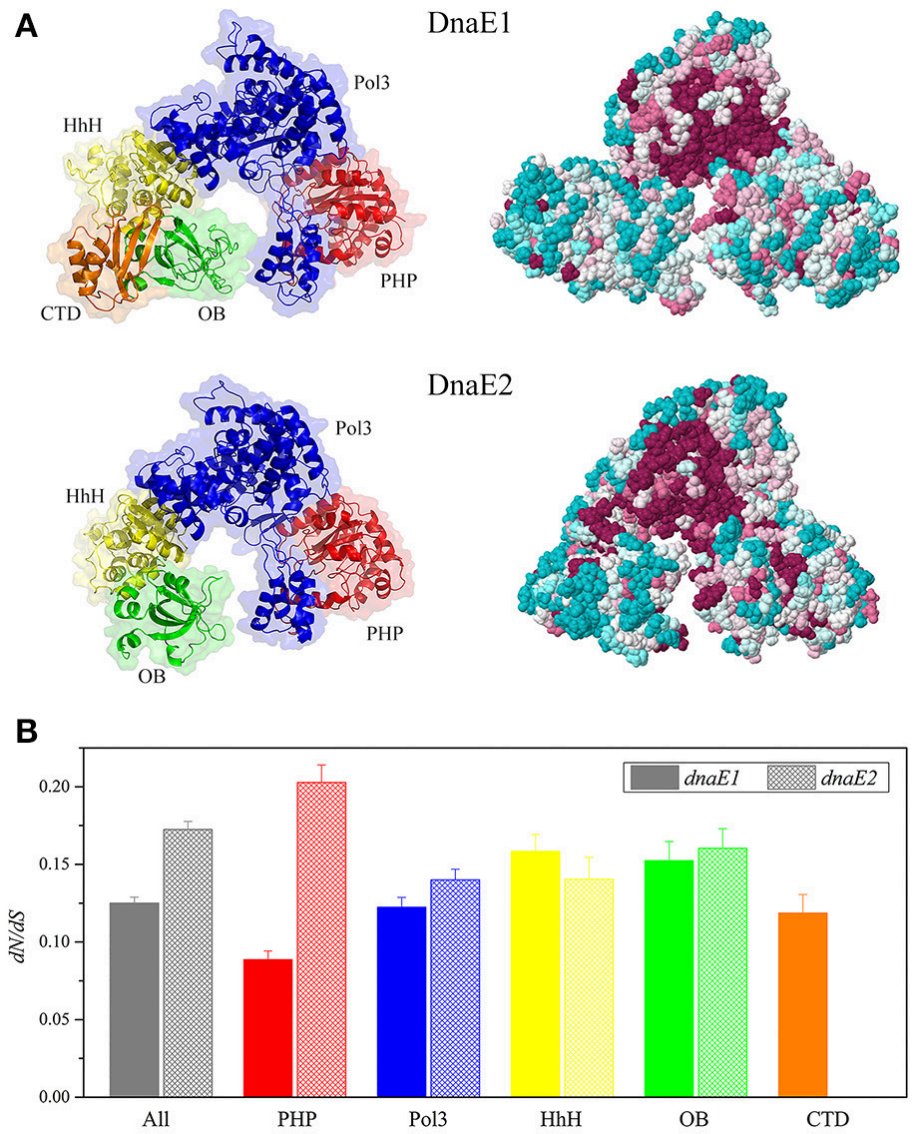
**Structure modeling of DK1622 DnaE1 and DnaE2 (A)** and comparison of the domain conservation of myxobacterial *dnaE1* and *dnaE2* **(B)**. **(A)** Three-dimensional structures showing different domains in different colors (left panels) and evolutionary conservation of surface amino acids (right panels); **(B)** The *dN/dS* values of *dnaE1* and *dnaE2* and the different domains of those sequenced myxobacteria.

Imbalanced effects of the deletion and overexpression of *dnaE2* suggested that the structural differences of the two DnaE proteins played more important roles for function than their expressions. Structural differences between DnaE2 and DnaE1 have been thoroughly investigated (Warner et al., 2010; McHenry, 2011a,b; Timinskas et al., 2014). To determine whether the essentiality of *dnaE1* was due to high expressions in DK1622, we retried to knock out the *dnaE1* gene in the *dnaE2*-overexpressing mutant (YL1605) using the pBJ113Cm-5844 plasmid but failed. This result indicated that *dnaE2* could not replace *dnaE1* even if the *dnaE2* gene was overexpressed, which confirmed that the non-fungibility of DnaE1 was due to the structural differences between DnaE1 and DnaE2. We modeled three-dimensional structures of DK1622 DnaE1 and DnaE2 (Figure 4A), using the threading approach (Yang et al., 2015). The estimated TM-scores are 0.84 ± 0.08 and 0.87 ± 0.07 for the modeled structures of DnaE1 and DnaE2, respectively. In general, the modeled protein structure with a TM-score > 0.5 is acceptable with the correct topology (Yang et al., 2015). The DnaE1 and DnaE2 proteins of *M. xanthus* DK1622 had highly similar three-dimensional structures, even though their sequences were highly different. The active sites and some of the DNA binding surfaces were also highly conserved in the DnaE1 and DnaE2 polymerases. In addition, the two DnaE proteins were also considerably conserved of their surface amino acid positions (Figure 4A, right panels). However, the DnaE1 polymerase contained five domains, including PHP, Pol3, HhH, OB, and CTD domains, while DnaE2 had four domains, lacking the CTD domain (Figure 4A, left panels), which is important for binding to Pol III τ-subunit (Liu et al., 2013).

In order to get insight into the domain conservation, we estimated the *dN/dS* values, an indicator of the selective pressure acting on a protein-coding gene, for the duplicated *dnaE* genes of those sequenced myxobacteria. The *dN/dS* values of the complete *dnaE1* and *dnaE2* sequences were 0.125 (95% confidence interval: 0.121–0.129) and 0.172 (95% confidence interval: 0.167–0.178), respectively, suggesting both polymerases were subjected to a strong negative selection pressure for their strict functional constraint. The *dN/dS* values were close between the Pol3, HhH, and OB domain sequences of the *dnaE1* and *dnaE2* genes, but had a great difference in the PHP sequence (*t*-test, *P* < 0.01, Figure 4B), which has a binding site of the proofreading ε-subunit (Wieczorek and McHenry, 2006; Ozawa et al., 2013). The *dN/dS* value of the *dnaE2* PHP domain was more than twice than that of *dnaE1*. Accordingly, *dnaE1* is more evolutionarily conservative than *dnaE2* in myxobacteria. Stano et al. reported that the PHP domain of DnaE-type polymerases had 3′-5′ exonuclease activity (Stano et al., 2006). Similarly, the PHP domain of Mtb DnaE1 retains all amino acids associated with exonuclease activity in *M. tuberculosis*, but the DnaE2 PHP lost some key residues (Wieczorek and McHenry, 2006; Baños et al., 2008). It was speculated that the loss of conserved residues in the DnaE2 PHP domain affected relative fidelity. The DNA polymerase III complexes constructed with the two DnaE proteins thus probably had divergent functions.

## Discussion

DNA polymerase III holoenzyme is the main polymerase for bacterial DNA replication. While most bacterial cells contain single copies of *dnaE*, encoding for the alpha subunit of the polymerase, some bacteria contain duplicate *dnaE* genes (Timinskas et al., 2014). There are several reports dealt with the functions of duplicated *dnaE* genes and have revealed some key characteristics of *dnaE2*, for example, the second *dnaE* gene encodes an error-prone enzyme and is dispensable. However, the function of the duplicate *dnaE* is still an enigma. For example, what is the role of the second copy of *dnaE* in bacteria, why do bacterial cells need an error-prone DNA polymerase enzyme, and how does the error-prone enzyme play functions in bacterial cells?

Myxobacteria have complex multicellular social behaviors, and all sequenced myxobacterial genomes contain duplicated *dnaE* genes. Given the high conservation in myxobacteria (Figure S1), the DnaE2 subunit should be important for myxobacterial cells. The expression of *dnaE1* was greatly higher than that of the *dnaE2* gene throughout the lifecycle of *M. xanthus* cells. Obviously, the *dnaE1* gene plays the major and essential role in chromosome replication in *M. xanthus* cells, not only in the growth stage but also in the development stage, thus ensuring the population genetically stable. In contrast, the *dnaE2* gene expressed approximately ten times lower than the *dnaE1* gene did in either the growth or the developmental stage, which is consistent with their weak cellular effects. Although weakly affected the growth and social motility, phenotypic characteristics of *dnaE2* mutants supported that *dnaE2* strongly affected the development progress. However, because the expression of *dnaE1* was also decreased, the deficiencies of development and sporulation in the *dnaE2* deletion mutant were the results of the synergetic effects of the two *dnaE* genes.

In nutrition-limited conditions, *M. xanthus* populations aggregate and develop fruiting bodies, which contain stress resistant myxospores (Shimkets, 1990; Shimkets et al., 2006). Unlike the formation progress of endospores in *Bacillus subtilis* (Errington and Wake, 1991), *M. xanthus* cells carry out a round of chromosome replication in the early stage of development (Tzeng and Singer, 2005). We demonstrated that the deletion of *dnaE2* caused obvious phenotypic deficiencies in development and sporulation. The development program of *M. xanthus* is a multicellular process requiring the coordinated expression of numerous regulation pathways to produce extracellular A- to E-signals and intracellular signals (Kaiser, 2004). Stringent response to the starvation of amino acids, the (p)ppGpp synthase enzyme (RelA) produces the chemical signal (p)ppGpp to initiate the development process (Singer and Kaiser, 1995; Harris et al., 1998). Rosario and Singer showed that inhibition of DNA replication within the first 6 h of development results in a block in developmental program, and the *dnaA* gene that encodes the initiator protein for DNA replication is not essential for development but allow for the proper timing and maximum efficiency of the sporulation process (Rosario and Singer, 2010). Our data improve the understanding of chromosome replication in the early stage of development of *M. xanthus*. We determined that, together with the essential DnaE1 polymerase, the error-prone DnaE2 enzyme participated in the chromosome replication of development process in *M. xanthus* DK1622, but in low fidelity for the genome replication due to its error-prone characteristic. It is known that suitable mutation rates can increase the adaptation abilities, whereas high mutation rates damage bacterial cells (Oliver et al., 2000; Giraud et al., 2001; Wielgoss et al., 2013). In new environments, the mutations probably increase for rapid adaptation, while in stable conditions, the mutations may decrease to reduce damages to cells. Thus, bacterial cells have to balance the mutation rates. The previous studies suggested that the bacterial land colonization is dominated by the emergence of *dnaE2*, followed by a series of niche-specific genomic adaptations, including GC content increase, intensive horizontal gene transfer and constant genome expansion (Wu et al., 2014). We suggested that the DnaE2 is rather a balancer to control the genome mutations to a suitable rate, based on its error-prone characteristic and its low expressions in either the growth or development stage. Low concentrations make the replication carried out by the DnaE2 polymerases occupy small fractions. We found that artificial overexpression of the *dnaE2* gene significantly increased the genome mutation rate, which suggested to be damaged for cell survivals. In addition, because the deletion of *dnaE2* caused deficiency of development and sporulation, the presence of the gene had some more effects on cellular behaviors. Using the master strategy, *M. xanthus* populations are able to live through the changing environments.

## Experimental procedures

### Strains and cultivation

The bacterial strains used in this study are listed in Table S2. The *M. xanthus* strains were cultivated in Casitone-based CTT medium (Hodgkin and Kaiser, 1977) for growth assays and on TPM agar plate (Kearns et al., 2000) for developmental assays. *E. coli* strains were routinely grown on Luria-Bertani (LB) agar or in LB liquid broth. *M. xanthus* strains were incubated at 30°C, and *E. coli* strains were at 37°C. When required, a final concentration of 40 μg/ml of kanamycin (Km) was added to the solid or liquid media.

### Construction of *M. xanthus* mutants

We performed the in-frame deletions of the *dnaE1, dnaE2, mutS*, and *mutL* genes in *M. xanthus* strains using pBJ113 plasmid, respectively. The plasmid contains a kanamycin resistant cassette for the first round of screening and a *galK* gene for the negative screening (Ueki et al., 1996). Briefly, homologous arms of *dnaE1, dnaE2, mutS*, or *mutL* were cloned with primers (listed in Table S3) and the products were inserted into the EcoRI/XbaI site of pBJ113. The resulting plasmid were introduced into *M. xanthus* DK1622 strains via electroporation (1.25 kV, 400 W, 25 mF, 0.1-cm cuvette gap), respectively. The second round of screening was then performed on CTT plates containing 1% galactose (Sigma). The deletion mutants that grew on galactose but were sensitive to kanamycin were identified and verified by PCR amplification and sequencing. Restriction enzymes, DNA ligase, and other DNA enzymes were used according to the manufacturers' recommendations. All fragments were validated by Sanger 3730 sequencing.

### Complementation and overexpression of *dnaE2* gene

The *dnaE2* gene and upstream 500 bp sequence that contains the whole promoter of *dnaE2* gene was cloned using the primer pair of MXAN_3982_NativeF and MXAN_3982_NativeR. The fragment and pSWU19 plasmid (replacing the *tet* resistant cassette of pSWU30 with a kanamycin resistant cassette) were digested with the XbaI/EcoRI digestion solution. The two fragments were ligated with T4 DNA ligase, producing the recombinant plasmid pSW3982. The promoter of the *pilA* gene was cloned using the primer pair of MXAN_3982P_UF and MXAN_3982P_UR, and fused with the *dnaE2* gene. The fusion product was inserted into XbaI/EcoRI sites of pSWU19, producing the plasmid pSWp3982.

The *dnaE2* fusion construct was transferred by electroporation into *M. xanthus* DK1622. Individual kanamycin-resistant clones were selected, and the mutants of the *dnaE2* transcription fusion were verified by PCR amplification (primers are listed in Table S3) and sequencing.

### Mutation rate assay

The mutation rate assay was conducted by screening the nalidixic acid resistant strains according to previous report (Tzeng et al., 2006). Briefly, approximately 5 × 10^9^ cells of *M. xanthus* strains were placed on CTT agar containing 40 μg/ml nalidixic acid. Nalidixic acid- resistant candidates were counted and mutation rate was calculated using the following formula, *r* = *N*_*m*_/*N*_0_, where *r* is the mutation rate, *N*_*m*_ represents the count of nalidixic acid resistant mutant, and *N*_*0*_ is the count of cells placed on the screening plate.

### Development and sporulation assays

Developmental experiments were performed on TPM agar plates (10 mM Tris [pH 7.6], 8 mM MgSO_4_, and 1 mM KH_2_PO_4_ containing 1.5% agar), as described below. Cells were allowed to develop in a humidity chamber at 30°C. When indicated, nalidixic acid solution of the 20 μg/ml concentration was added onto different plates at the time of 0, 6, 12, 18, or 24 h for 1 h, and then the extra solution was poured out. After 120 h cultivation at 30°C. Sporulation was conducted as follows: five colonies were collected and resuspended in liquid CTT media. Sonication was conducted at 200 W for 4 s twice to release myxospores from fruiting bodies. The myxospore suspensions were incubated at 55°C for 2 h to kill the vegetative cells. Then the suspensions were serially diluted and aliquots of 50 μL were used to plate. The sporulation rate was counted after 5 days of cultivation on the CTT plate.

### RNA extraction and quantitative real-time PCR assay

The *M. xanthus* DK1622 cells were concentrated to approximately 1.75 × 10^10^ cells per milliliter. The cell suspension was washed three times using TPM buffer. RNA was extracted immediately with a BIOZOL total RNA extraction reagent (BioFlux) following the manufacturer's instructions. Genomic DNA contamination was removed by using DNA eraser supplied in the PrimeScript RT Reagent kit with gDNA Eraser (TaKaRa). The purified RNA extracts were transcribed reversely to cDNA and stored in aliquots at −80°C. Quantitative real-time PCR was performed in a total reaction volume of 25 μl, containing 250 nM primers, 12.5 μl of SYBR Premix Ex Taq GC mix (TaKaRa), 10.5 μl of RNase-free water (TaKaRa), and 1 μl of a 10-fold-diluted cDNA template. PCR was conducted in a Roche LightCycler 480 sequence detection system, following the program: 3 min at 95°C, followed by 40 cycles of 30 s at 95°C, 30 s at 55°C, and 15 s at 72°C. The *gapA* gene, encoding for glyceraldehyde-3-phosphate dehydrogenase, was used as the normalization signal. Calibration curves of *gapA, MXAN_3982*, and *MXAN_5844* were generated from a 10-fold dilution of *M. xanthus* DK1622 genomic DNA. The primer pairs used for each gene are listed in Table S3.

### Bioinformatics analyses

The *dnaE1* and *dnaE2* genes were extracted from the genome of *M. xanthus* DK1622 strain. All the *dnaE* genes used for phylogenetic analysis were extracted according to the Genomic Encyclopedia of Bacteria and Archaea (Wu et al., 2009). These genes were translated into amino acid sequences according to the bacterial standard codon. A multiple sequence alignment of the DnaE1 and DnaE2 proteins was established using the MAFFT program (Katoh and Standley, 2013). The phylogenetic tree was constructed using the PhyML program with the LG substitution model and the four substitution rate categories (Guindon et al., 2010). LG model was regarded as the best substitution model by automatic model selection. Branch support was calculated using the approximate likelihood ratio tests (aLRT SH-like) (Anisimova and Gascuel, 2006). The phylogenetic tree was visualized by iTOL (Letunic and Bork, 2016).

The structures of the DnaE1 and DnaE2 were modeled using the I-TASSER program based on a threading approach (Yang et al., 2015). The evolutionary conservation of amino acid positions in the *dnaE1* and *dnaE2* sequences was estimated by using the ConSurf algorithm (Ashkenazy et al., 2010). The JTT substitution matrix was used and the computation was based on the empirical Bayesian paradigm. The conservation scale was defined from the most variable amino acid positions (grade 1), which were considered to be evolved rapidly, to the most conservative positions (grade 9), which were considered to be evolved slowly. The sequences and modeled structures of the DnaE1 and DnaE2 were shown in nine-color conservation grades.

The number ratio of nonsynonymous substitutions per nonsynonymous site (*dN*) to synonymous substitutions per synonymous site (*dS*), *dN/dS*, is an indicator of the selective pressure acting on a protein-coding gene. The *dN/dS* calculation of the *dnaE1* and *dnaE2* locus was set at the 95% confidence intervals, using the Datamonkey web server (Delport et al., 2010). For the calculation, a multiple sequence alignment of the nucleic acid sequences of myxobacterial *dnaE1* and *dnaE2* genes, based on codon, was established with TranslatorX (Abascal et al., 2010). Domains of DnaE1 and DnaE2 proteins were extracted by blasting against the PFAM database (Finn et al., 2014).

The statistical analysis was conducted using IBM SPSS Statistics.

## Author contributions

Conceived and designed the experiments: YL and RP. Performed the experiments and analyzed the data: RP, JC, WF, ZZ, JY, and ZL. Wrote the paper: YL and RP.

### Conflict of interest statement

The authors declare that the research was conducted in the absence of any commercial or financial relationships that could be construed as a potential conflict of interest.

## Supplementary material

Figure S1 **Phylogenetic analyses of myxobacterial DnaE protein sequences**. Some determined DnaE1 and DnaE2 proteins from other bacteria species were also included in the tree.

Table S1 **Information of dnaE genes in sequenced myxobacterial genomes**.

Table S2 **Strains and plasmids used in this study**.

Table S3 **Primers used in this study**.

## References

[B1] Abascal F. Zardoya R. Telford M. J. (2010). TranslatorX: multiple alignment of nucleotide sequences guided by amino acid translations. Nucleic Acids Res 38, W7–W13. 10.1093/nar/gkq291 20435676 PMC2896173

[B2] Abella M. Erill I. Jara M. Mazón G. Campoy S. Barbé J. (2004). Widespread distribution of a lexA-regulated DNA damage-inducible multiple gene cassette in the Proteobacteria phylum. Mol. Microbiol. 54, 212–222. 10.1111/j.1365-2958.2004.04260.x 15458417

[B3] Anisimova M. Gascuel O. (2006). Approximate likelihood-ratio test for branches: a fast, accurate, and powerful alternative. Syst. Biol. 55, 539–552. 10.1080/10635150600755453 16785212

[B4] Ashkenazy H. Erez E. Martz E. Pupko T. Ben-Tal N. (2010). ConSurf 2010: calculating evolutionary conservation in sequence and structure of proteins and nucleic acids. Nucleic Acids Res. 38, W529–W533. 10.1093/nar/gkq399 20478830 PMC2896094

[B5] Baños B. Lázaro J. M. Villar L. Salas M. De Vega M. (2008). Editing of misaligned 3'-termini by an intrinsic 3'-5' exonuclease activity residing in the PHP domain of a family X DNA polymerase. Nucleic Acids Res. 36, 5736–5749. 10.1093/nar/gkn526 18776221 PMC2566882

[B6] Boshoff H. I. Reed M. B. Barry C. E. III Mizrahi V. (2003). DnaE2 polymerase contributes to *in vivo* survival and the emergence of drug resistance in *Mycobacterium tuberculosis*. Cell 113, 183–193. 10.1016/S0092-8674(03)00270-8 12705867

[B7] Brinkhoff T. Fischer D. Vollmers J. Voget S. Beardsley C. Thole S.. (2012). Biogeography and phylogenetic diversity of a cluster of exclusively marine myxobacteria. ISME J. 6, 1260–1272. 10.1038/ismej.2011.190 22189493 PMC3358034

[B8] Delport W. Poon A. F. Frost S. D. Kosakovsky Pond S. L. (2010). Datamonkey 2010: a suite of phylogenetic analysis tools for evolutionary biology. Bioinformatics 26, 2455–2457. 10.1093/bioinformatics/btq429 20671151 PMC2944195

[B9] Dworkin M. (1996). Recent advances in the social and developmental biology of the myxobacteria. Microbiol. Rev. 60, 70–102. 8852896 10.1128/mr.60.1.70-102.1996 PMC239419

[B10] Errington J. Wake R. G. (1991). Chromosome strand segregation during sporulation in *Bacillus subtilis*. Mol. Microbiol. 5, 1145–1149. 10.1111/j.1365-2958.1991.tb01887.x 1956292

[B11] Evans R. J. Davies D. R. Bullard J. M. Christensen J. Green L. S. Guiles J. W.. (2008). Structure of PolC reveals unique DNA binding and fidelity determinants. Proc. Natl. Acad. Sci. U.S.A. 105, 20695–20700. 10.1073/pnas.0809989106 19106298 PMC2634937

[B12] Finn R. D. Bateman A. Clements J. Coggill P. Eberhardt R. Y. Eddy S. R.. (2014). Pfam: the protein families database. Nucleic Acids Res. 42, D222–D230. 10.1093/nar/gkt1223 24288371 PMC3965110

[B13] Giraud A. Matic I. Tenaillon O. Clara A. Radman M. Fons M.. (2001). Costs and benefits of high mutation rates: adaptive evolution of bacteria in the mouse gut. Science 291, 2606–2608. 10.1126/science.1056421 11283373

[B14] Goldman B. S. Nierman W. C. Kaiser D. Slater S. C. Durkin A. S. Eisen J. A.. (2006). Evolution of sensory complexity recorded in a myxobacterial genome. Proc. Natl. Acad. Sci. U.S.A. 103, 15200–15205. 10.1073/pnas.0607335103 17015832 PMC1622800

[B15] Guindon S. Dufayard J. F. Lefort V. Anisimova M. Hordijk W. Gascuel O. (2010). New algorithms and methods to estimate maximum-likelihood phylogenies: assessing the performance of PhyML 3.0. Syst. Biol. 59, 307–321. 10.1093/sysbio/syq010 20525638

[B16] Harris B. Z. Kaiser D. Singer M. (1998). The guanosine nucleotide (p)ppGpp initiates development and A-factor production in *Myxococcus xanthus*. Genes Dev. 12, 1022–1035. 10.1101/gad.12.7.1022 9531539 PMC316683

[B17] Hodgkin J. Kaiser D. (1977). Cell-to-cell stimulation of movement in nonmotile mutants of Myxococcus. Proc. Natl. Acad. Sci. U.S.A. 74, 2938–2942. 10.1073/pnas.74.7.2938 16592422 PMC431354

[B18] Ito J. Braithwaite D. K. (1991). Compilation and alignment of DNA polymerase sequences. Nucleic Acids Res. 19, 4045–4057. 10.1093/nar/19.15.4045 1870963 PMC328540

[B19] Jiang D. M. Kato C. Zhou X. W. Wu Z. H. Sato T. Li Y. Z. (2010). Phylogeographic separation of marine and soil myxobacteria at high levels of classification. ISME J. 4, 1520–1530. 10.1038/ismej.2010.84 20596070

[B20] Kaiser D. (2004). Signaling in myxobacteria. Annu. Rev. Microbiol. 58, 75–98. 10.1146/annurev.micro.58.030603.123620 15487930

[B21] Kaiser D. Losick R. (1993). How and why bacteria talk to each other. Cell 73, 873–885. 10.1016/0092-8674(93)90268-U 8500179

[B22] Katoh K. Standley D. M. (2013). MAFFT multiple sequence alignment software version 7: improvements in performance and usability. Mol. Biol. Evol. 30, 772–780. 10.1093/molbev/mst010 23329690 PMC3603318

[B23] Kearns D. B. Campbell B. D. Shimkets L. J. (2000). *Myxococcus xanthus* fibril appendages are essential for excitation by a phospholipid attractant. Proc. Natl. Acad. Sci. U.S.A. 97, 11505–11510. 10.1073/pnas.210448597 11016978 PMC17230

[B24] Kelman Z. O'donnell M. (1995). DNA polymerase III holoenzyme: structure and function of a chromosomal replicating machine. Annu. Rev. Biochem. 64, 171–200. 10.1146/annurev.bi.64.070195.001131 7574479

[B25] Koorits L. Tegova R. Tark M. Tarassova K. Tover A. Kivisaar M. (2007). Study of involvement of ImuB and DnaE2 in stationary-phase mutagenesis in Pseudomonas putida. DNA Repair (Amst). 6, 863–868. 10.1016/j.dnarep.2007.01.010 17331811

[B26] Lamers M. H. Georgescu R. E. Lee S. G. O'donnell M. Kuriyan J. (2006). Crystal structure of the catalytic α subunit of *E. coli* replicative DNA polymerase III. Cell 126, 881–892. 10.1016/j.cell.2006.07.028 16959568

[B27] Letunic I. Bork P. (2016). Interactive tree of life (iTOL) v3: an online tool for the display and annotation of phylogenetic and other trees. Nucleic Acids Res. 44, W242–W245. 10.1093/nar/gkw290 27095192 PMC4987883

[B28] Li S. G. Zhou X. W. Li P. F. Han K. Li W. Li Z. F.. (2012). The existence and diversity of myxobacteria in lake mud - a previously unexplored myxobacteria habitat. Environ. Microbiol. Rep. 4, 587–595. 10.1111/j.1758-2229.2012.00373.x 23760929

[B29] Liu B. Lin J. Steitz T. A. (2013). Structure of the PolIIIα-tauc-DNA complex suggests an atomic model of the replisome. Structure 21, 658–664. 10.1016/j.str.2013.02.002 23478062 PMC3652607

[B30] McHenry C. S. (2011a). Bacterial replicases and related polymerases. Curr. Opin. Chem. Biol. 15, 587–594. 10.1016/j.cbpa.2011.07.018 21855395 PMC3190588

[B31] McHenry C. S. (2011b). Breaking the rules: bacteria that use several DNA polymerase IIIs. EMBO Rep. 12, 408–414. 10.1038/embor.2011.51 21475246 PMC3090020

[B32] Oliver A. Cantón R. Campo P. Baquero F. Blázquez J. (2000). High frequency of hypermutable *Pseudomonas aeruginosa* in cystic fibrosis lung infection. Science 288, 1251–1254. 10.1126/science.288.5469.1251 10818002

[B33] Ozawa K. Horan N. P. Robinson A. Yagi H. Hill F. R. Jergic S.. (2013). Proofreading exonuclease on a tether: the complex between the *E. coli* DNA polymerase III subunits α, ε, θ, and β reveals a highly flexible arrangement of the proofreading domain. Nucleic Acids Res. 41, 5354–5367. 10.1093/nar/gkt162 23580545 PMC3664792

[B34] Reichenbach H. (1999). The ecology of the myxobacteria. Environ. Microbiol. 1, 15–21. 10.1046/j.1462-2920.1999.00016.x 11207714

[B35] Robinson A. Causer R. J. Dixon N. E. (2012). Architecture and conservation of the bacterial DNA replication machinery, an underexploited drug target. Curr. Drug Targets 13, 352–372. 10.2174/138945012799424598 22206257 PMC3290774

[B36] Rosario C. J. Singer M. (2010). Developmental expression of dnaA is required for sporulation and timing of fruiting body formation in *Myxococcus xanthus*. Mol. Microbiol. 76, 1322–1333. 10.1111/j.1365-2958.2010.07178.x 20487266

[B37] Shimkets L. J. (1990). Social and developmental biology of the myxobacteria. Microbiol. Rev. 54, 473–501. 1708086 10.1128/mr.54.4.473-501.1990 PMC372790

[B38] Shimkets L. J. Dworkin M. Reichenbach H. (2006). The myxobacteria, in The prokaryotes, eds Dworkin M. Falkow S. Rosenberg E. Schleifer K. H. Stackebrandt E.(New York, NY: Springer), 31–115.

[B39] Singer M. Kaiser D. (1995). Ectopic production of guanosine penta- and tetraphosphate can initiate early developmental gene expression in *Myxococcus xanthus*. Genes Dev. 9, 1633–1644. 10.1101/gad.9.13.1633 7628697

[B40] Sniegowski P. D. Gerrish P. J. Lenski R. E. (1997). Evolution of high mutation rates in experimental populations of *E. coli*. Nature 387, 703–705. 10.1038/42701 9192894

[B41] Stano N. M. Chen J. McHenry C. S. (2006). A coproofreading Zn 2^+^-dependent exonuclease within a bacterial replicase. Nat. Struct. Mol. Biol. 13, 458–459. 10.1038/nsmb1078 16604084

[B42] Timinskas K. Balvociute M. Timinskas A. Venclovas C. (2014). Comprehensive analysis of DNA polymerase III α subunits and their homologs in bacterial genomes. Nucleic Acids Res. 42, 1393–1413. 10.1093/nar/gkt900 24106089 PMC3919608

[B43] Tsai H. H. Shu H. W. Yang C. C. Chen C. W. (2012). Translesion-synthesis DNA polymerases participate in replication of the telomeres in Streptomyces. Nucleic Acids Res. 40, 1118–1130. 10.1093/nar/gkr856 22006845 PMC3273824

[B44] Tzeng L. Ellis T. N. Singer M. (2006). DNA replication during aggregation phase is essential for *Myxococcus xanthus* development. J. Bacteriol. 188, 2774–2779. 10.1128/JB.188.8.2774-2779.2006 16585738 PMC1447012

[B45] Tzeng L. Singer M. (2005). DNA replication during sporulation in *Myxococcus xanthus* fruiting bodies. Proc. Natl. Acad. Sci. U.S.A. 102, 14428–14433. 10.1073/pnas.0506969102 16183740 PMC1228275

[B46] Ueki T. Inouye S. Inouye M. (1996). Positive-negative KG cassettes for construction of multi-gene deletions using a single drug marker. Gene 183, 153–157. 10.1016/S0378-1119(96)00546-X 8996101

[B47] Warner D. F. Ndwandwe D. E. Abrahams G. L. Kana B. D. Machowski E. E. Venclovas C.. (2010). Essential roles for imuA'- and imuB-encoded accessory factors in DnaE2-dependent mutagenesis in *Mycobacterium tuberculosis*. Proc. Natl. Acad. Sci. U.S.A. 107, 13093–13098. 10.1073/pnas.1002614107 20615954 PMC2919956

[B48] Wieczorek A. McHenry C. S. (2006). The NH2-terminal php domain of the α subunit of the *Escherichia coli* replicase binds the epsilon proofreading subunit. J. Biol. Chem. 281, 12561–12567. 10.1074/jbc.M513844200 16517598

[B49] Wielgoss S. Barrick J. E. Tenaillon O. Wiser M. J. Dittmar W. J. Cruveiller S.. (2013). Mutation rate dynamics in a bacterial population reflect tension between adaptation and genetic load. Proc. Natl. Acad. Sci. U.S.A. 110, 222–227. 10.1073/pnas.1219574110 23248287 PMC3538217

[B50] Wu D. Hugenholtz P. Mavromatis K. Pukall R. Dalin E. Ivanova N. N.. (2009). A phylogeny-driven genomic encyclopaedia of Bacteria and Archaea. Nature 462, 1056–1060. 10.1038/nature08656 20033048 PMC3073058

[B51] Wu H. Fang Y. Yu J. Zhang Z. (2014). The quest for a unified view of bacterial land colonization. ISME J. 8, 1358–1369. 10.1038/ismej.2013.247 24451209 PMC4069389

[B52] Wu S. S. Kaiser D. (1995). Genetic and functional evidence that Type IV pili are required for social gliding motility in *Myxococcus xanthus*. Mol. Microbiol. 18, 547–558. 10.1111/j.1365-2958.1995.mmi_18030547.x 8748037

[B53] Yang J. Yan R. Roy A. Xu D. Poisson J. Zhang Y. (2015). The I-TASSER Suite: protein structure and function prediction. Nat. Methods 12, 7–8. 10.1038/nmeth.3213 25549265 PMC4428668

[B54] Zhao X. Zhang Z. Yan J. Yu J. (2007). GC content variability of eubacteria is governed by the pol III α subunit. Biochem. Biophys. Res. Commun. 356, 20–25. 10.1016/j.bbrc.2007.02.109 17336933

[B55] Zhou X. W. Li S. G. Li W. Jiang D. M. Han K. Wu Z. H.. (2014). Myxobacterial community is a predominant and highly diverse bacterial group in soil niches. Environ. Microbiol. Rep. 6, 45–56. 10.1111/1758-2229.12107 24596262

