# Supplementary figures and images for: Error-prone DnaE2 Balances the Genome Mutation Rates in Myxococcus xanthus DK1622
Source: Front Microbiol. 2017 Feb 1;8:122. doi: 10.3389/fmicb.2017.00122 (PMC5285347; doi:10.3389/fmicb.2017.00122)

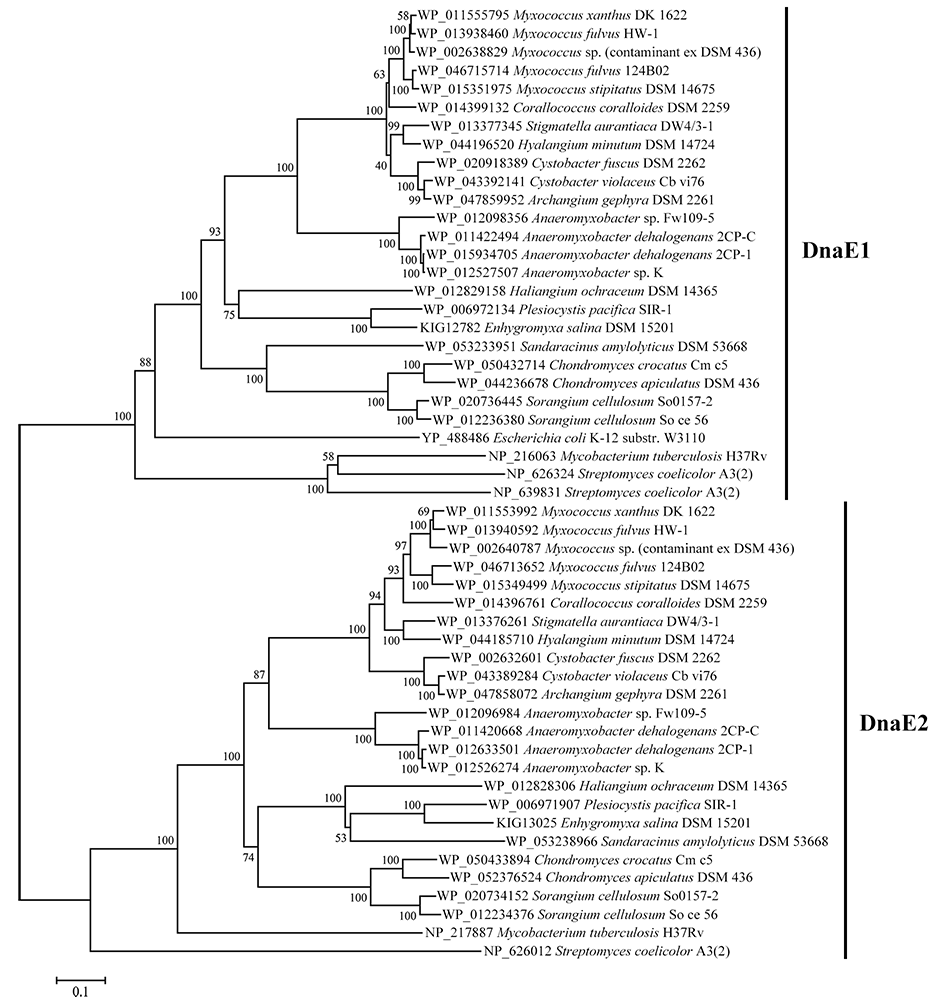

Supplement: Figure S1 — Phylogenetic analyses of myxobacterial DnaE protein sequences. Some determined DnaE1 and DnaE2 proteins from other bacteria species were also included in the tree. [file Image1.TIF]
