# Supplementary material for: Error-prone DnaE2 Balances the Genome Mutation Rates in Myxococcus xanthus DK1622
Source: Front Microbiol. 2017 Feb 1;8:122. doi: 10.3389/fmicb.2017.00122 (PMC5285347; doi:10.3389/fmicb.2017.00122)
Supplement: Table S1 — Information of dnaE genes in sequenced myxobacterial genomes. [file Table1.DOCX]

**Table S1. Information of *dnaE* genes in sequenced myxobacterial genomes.**

| **Strain** | **Genome Size (Mb)** | **GC %** | **Gene name** | **Locus tag** | **Length (bp)** | **Protein product** |
| --- | --- | --- | --- | --- | --- | --- |
| *Myxococcus xanthus* DK1622 | 9.14 | 68.9 | *dnaE1* | MXAN_5844 | 3558 | WP_011555795 |
|  |  |  | *dnaE2* | MXAN_3982 | 3042 | WP_011553992 |
| *Myxococcus fulvus* HW-1 | 9.00 | 70.6 | *dnaE1* | LILAB_17045 | 3555 | WP_013938460 |
|  |  |  | *dnaE2* | LILAB_28025 | 3045 | WP_013940592 |
| *Myxococcus* sp. (contaminant ex DSM 436) | 9.49 | 69.2 | *dnaE1* | A176_001062 | 3555 | WP_002638829 |
|  |  |  | *dnaE2* | A176_002938 | 3084 | WP_002640787 |
| *Myxococcus fulvus* 124B02 | 11.05 | 70.0 | *dnaE1* | MFUL124B02_33955 | 3552 | WP_046715714 |
|  |  |  | *dnaE2* | MFUL124B02_21270 | 3069 | WP_046713652 |
| *Myxococcus stipitatus* DSM 14675 | 10.35 | 69.2 | *dnaE1* | MYSTI_06448 | 3552 | WP_015351975 |
|  |  |  | *dnaE2* | MYSTI_03933 | 3078 | WP_015349499 |
| *Corallococcus coralloides* DSM 2259 | 10.08 | 69.9 | *dnaE1* | COCOR_06344 | 3546 | WP_014399132 |
|  |  |  | *dnaE2* | COCOR_03949 | 3015 | WP_014396761 |
| *Stigmatella aurantiaca* DW4/3-1 | 10.26 | 67.5 | *dnaE1* | STAUR_6511 | 3558 | WP_013377345 |
|  |  |  | *dnaE2* | STAUR_4452 | 3024 | WP_013376261 |
| *Hyalangium minutum* DSM 14724 | 11.19 | 68.0 | *dnaE1* | DB31_2972 | 3558 | WP_044196520 |
|  |  |  | *dnaE2* | DB31_5577 | 3024 | WP_044185710 |
| *Archangium gephyra* DSM 2261 | 12.49 | 69.4 | *dnaE1* | AA314_08368 | 3573 | WP_047859952 |
|  |  |  | *dnaE2* | AA314_05821 | 3036 | WP_047858072 |
| *Cystobacter violaceus* Cb vi76 | 12.54 | 68.9 | *dnaE1* | Q664_08995 | 3576 | WP_043392141 |
|  |  |  | *dnaE2* | Q664_01865 | 3030 | WP_043389284 |
| *Cystobacter fuscus* DSM 2262 | 12.28 | 68.6 | *dnaE1* | D187_003548 | 3570 | WP_020918389 |
|  |  |  | *dnaE2* | D187_004295 | 3036 | WP_002632601 |
| *Anaeromyxobacter dehalogenans* 2CP-1 | 5.03 | 74.7 | *dnaE1* | A2cp1_3593 | 3633 | WP_015934705 |
|  |  |  | *dnaE2* | A2cp1_2336 | 3429 | WP_012633501 |
| *Anaeromyxobacter dehalogenans* 2CP-C | 5.01 | 74.9 | *dnaE1* | Adeh_3445 | 3633 | WP_011422494 |
|  |  |  | *dnaE2* | Adeh_1612 | 3399 | WP_011420668 |
| *Anaeromyxobacter* sp. K | 5.06 | 74.8 | *dnaE1* | AnaeK_3525 | 3633 | WP_012527507 |
|  |  |  | *dnaE2* | AnaeK_2248 | 3402 | WP_012526274 |
| *Anaeromyxobacter* sp. Fw109-5 | 5.28 | 73.5 | *dnaE1* | Anae109_3552 | 3654 | WP_012098356 |
|  |  |  | *dnaE2* | Anae109_2200 | 3594 | WP_012096984 |
| *Haliangium ochraceum* DSM 14365 | 9.45 | 69.5 | *dnaE1* | Hoch_4061 | 3525 | WP_012829158 |
|  |  |  | *dnaE2* | Hoch_3204 | 3225 | WP_012828306 |
| *Plesiocystis pacifica* SIR-1 | 10.59 | 70.7 | *dnaE1* | PPSIR1_12278 | 3768 | WP_006972134 |
|  |  |  | *dnaE2* | PPSIR1_07248 | 3378 | WP_006971907 |
| *Enhygromyxa salina* DSM 15201 | 10.44 | 67.4 | *dnaE1* | DB30_01043 | 3795 | KIG12782 |
|  |  |  | *dnaE2* | DB30_00799 | 3360 | KIG13025 |
| *Sandaracinus amylolyticus* DSM 53668 | 10.33 | 72.0 | *dnaE1* | DB32_003932 | 3606 | WP_053233951 |
|  |  |  | *dnaE2* | DB32_005420 | 3189 | WP_053238966 |
| *Chondromyces crocatus* Cm c5 | 11.39 | 68.7 | *dnaE1* | CMC5_049860 | 3630 | WP_050432714 |
|  |  |  | *dnaE2* | CMC5_064550 | 3270 | WP_050433894 |
| *Chondromyces apiculatus* DSM 436 | 11.58 | 70.3 | *dnaE1* | CAP_6905 | 3612 | WP_044236678 |
|  |  |  | *dnaE2* | CAP_7840 | 3318 | WP_052376524 |
| *Sorangium cellulosum* So0157-2 | 14.78 | 72.1 | *dnaE1* | SCE1572_22570 | 3639 | WP_020736445 |
|  |  |  | *dnaE2* | SCE1572_10870 | 3360 | WP_020734152 |
| *Sorangium cellulosum* 'So ce 56' | 13.03 | 71.4 | *dnaE1* | sce3750 | 3639 | WP_012236380 |
|  |  |  | *dnaE2* | sce1741 | 3327 | WP_012234376 |
