# Supplementary material for: Error-prone DnaE2 Balances the Genome Mutation Rates in Myxococcus xanthus DK1622
Source: Front Microbiol. 2017 Feb 1;8:122. doi: 10.3389/fmicb.2017.00122 (PMC5285347; doi:10.3389/fmicb.2017.00122)
Supplement: Table S2 — Strains and plasmids used in this study. [file Table2.DOCX]

**Table S2. Strains and plasmids used in this study.**

| **Strains or plasmids** | | **Genotype or description** | **Source or references** |
| --- | --- | --- | --- |
|  | **Strains** |  |  |
|  | *M. xanthus* |  |  |
|  | DK1622 | Wild-type strains | D. Kaiser, University of Stanford |
|  | YL1601 | DK1622 Δ*MXAN_3982* (*dnaE2*) | This study |
|  | YL1602 | DK1622 Δ*MXAN_3897* (*mutS*) | This study |
|  | YL1603 | DK1622 Δ*MXAN_4026* (*mutL*) | This study |
|  | YL1604 | DK1622::pSWU19 | This study |
|  | YL1605 | DK1622::pSWp3982 (*dnaE2* with *pilA* promoter integrated at *attB* site) | This study |
|  | YL1606 | YL1501::pSW3982 (*dnaE2* with native promoter integrated at *attB* site) | This study |
|  |  |  |  |
|  | *E. coli* |  |  |
|  | DH5α(λpir) | ϕ80 *lacZ*Δ*M15* Δ*lacU169* *recA1 endA1*  *hsdR17 supE44 thi-1 gyrA relA1 λpir* | H.B. Kaplan, University of Texas |
|  | XL1-Blue MR | Δ(*mcrA*)183Δ(*mcrCB-hsdSMR-mrr*)*173 endA1 supE44 thi-1 recA1*  *gyrA96 relA1 lac* | Stratagene |
|  |  |  |  |
|  | **Plasmids** |  |  |
|  | pBJ113 | Gene replacement vector with KG cassette, Km^r^ | Z.M. Yang, Virginia Tech |
|  | pSWU30 | Site-specific integration vector with Mx8 attP integration site, Tet^r^ | Mignot Tâm, CNRS(Centre national de la recherché scientifique) |
|  | pSWU19 | Site-specific integration vector with Mx8 attP integration site, Kan^r^ | ([Wu & Kaiser, 1995](#_ENREF_1)) |
|  | pSW3982 | 3039bp fragment of *MXAN_3982*, with its upstream 511bp promoter sequence, inserted into XbaI/EcoRI sites of pSWU19, Kan^r^ | This study |
|  | pSWp3982 | 3039bp fragment of *MXAN_3982*, with 500bp *pilA* promoter, inserted into XbaI/EcoRI sites of pSWU19, Kan^r^ | This study |
|  | pBJ3982 | Upstream and downstream homologous arms of DK1622 *MXAN_3982* inserted into XbaI/EcoRI site of pBJ113, Km^r^ | This study |
|  | pBJ5844 | Upstream and downstream homologous arms of DK1622 *MXAN_5844* inserted into XbaI/EcoRI site of pBJ113, Km^r^ | This study |
|  | pBJ113Cm-5844 | Upstream and downstream homologous arms of DK1622 MXAN_5844 inserted into XbaI/EcoRI site of pBJ113Cm, Cm^r^ | This study |
|  | pBJ3897 | Upstream and downstream homologous arms of DK1622 *MXAN_3897* inserted into XbaI/EcoRI site of pBJ113, Km^r^ | This study |
|  | pBJ4026 | Upstream and downstream homologous arms of DK1622 *MXAN_4026* inserted into XbaI/EcoRI site of pBJ113, Km^r^ | This study |
