# Supplementary material for: Error-prone DnaE2 Balances the Genome Mutation Rates in Myxococcus xanthus DK1622
Source: Front Microbiol. 2017 Feb 1;8:122. doi: 10.3389/fmicb.2017.00122 (PMC5285347; doi:10.3389/fmicb.2017.00122)
Supplement: Table S3 — Primers used in this study. [file Table3.DOCX]

**Table S3. Primers used in this study.**

| **Primer name** | **Primer sequence (5’-3’)** |
| --- | --- |
| MXAN3897_UF | GCTCTAGAGCATGAACAGGCCCAGCACCACG |
| MXAN3897_UR | GTCCCAGGCGGGCGCGCCCGGCCCCACCTCGGGCGTC |
| MXAN3897_DF | GACGCCCGAGGTGGGGCCGGGCGCGCCCGCCTGGGAC |
| MXAN3897_DR | CCGAAGCTTCACCGGACGATCCTTGCTCACGCAC |
| MXAN4026_UF | GCTCTAGACGGCTTCTTCTTCGTCACCGTCTCC |
| MXAN4026_UR | CGGGGCACTCAGCGGGGGCGGCCGGACACCCTCC |
| MXAN4026_DF | CGGAGGGTGTCCGGCCGCCCCCGCTGAGTGCCCCGAAAATTGAC |
| MXAN4026_DR | CCGAAGCTTAGGAGTTCTCCCGGTCATGCGTCG |
| MXAN_3982_UF | GGAATTCCTGACGACGGGACTGGTGG |
| MXAN_3982_UR | GTTCGGTATCTAGATCCGGGCTGACCGCCGACCTCGAATG |
| MXAN_3982_DF | CCATTCGAGGTCGGCGGTCAGCCCGGATCTAGATACCGAACAGG |
| MXAN_3982_DR | CCCAAGCTTCTGCTGGCGGTCTTGTAG |
| MXAN_5844_UF | GGAATTCATTGGCGCAATCAACGCCCTC |
| MXAN_5844_UR | GGCCTCCAGGAGCGGAACGCGCATGCCGGGGCTGATTTCTAAAAGC |
| MXAN_5884_DF | CTTTTAGAAATCAGCCCCGGCATGCGCGTTCCGCTCCTG |
| MXAN_5884_DR | CCCAAGCTTTCCGCTTTGCGTAGTCGGTG |
| MXAN_3982P_UF | ACCAGCTCGGCGTAGTCCACGGGGGTCCTCAGAGAAGGTTG |
| MXAN_3982P_UR | CCGGAATTCTGGGGCTATTGGAGCGCTATC |
| MXAN_3982P_NR | CAACCTTCTCTGAGGACCCCCGTGGACTACGCCGAGCTGG |
| MXAN_3982_NativeF | TGCTCTAGATCACCCAGGCAGCGTACC |
| MXAN_3982_NativeR | CCGGAATTCCTCGCCATTGATGCGCACC |
| MXAN3982_F870 | GAAGCAGATTGAGCACGAGC |
| MXAN3982_R1106 | ATGTCTGGCGGTTCCTTG |
| MXAN5844_F149 | TCTACAAGAAGGCGAAGGAC |
| MXAN5844_R289 | CGTAACCCTCCTCGTTCTTC |
| gapA-F | GCCCTGGAAGAGCCTGAACG |
| gapA-R | TGGAGACGATGTGGTGCTTGG |
